# Macrolide therapy in cryptogenic organizing pneumonia: A case report and literature review

**DOI:** 10.3892/etm.2015.2183

**Published:** 2015-01-15

**Authors:** QUN-LI DING, DAN LV, BI-JIONG WANG, QIAO-LI ZHANG, YI-MING YU, SHI-FANG SUN, ZHONG-BO CHEN, HONG-YING MA, ZAI-CHUN DENG

**Affiliations:** Department of Respiratory Medicine, Affiliated Hospital, School of Medicine, Ningbo University, Ningbo, Zhejiang 315020, P.R. China

**Keywords:** macrolide, organizing pneumonia, treatment, inflammation, glucocorticoid, azithromycin

## Abstract

Cryptogenic organizing pneumonia (COP) is a pulmonary disorder associated with nonspecific clinical presentations. The macrolide class of antimicrobial agents is widely used to treat infectious and inflammatory respiratory diseases in humans. The present study reports a case of COP that was effectively treated with azithromycin in combination with glucocorticoid. A literature review of similar cases is also presented. It was found that all COP patients in the literature received macrolide treatment, including six cases with unknown clinical outcomes. For the remaining 29 patients, 20 patients initially received the macrolide as a single therapy and 4/5 of them (16 cases) were cured with a treatment time of 3–14 months, while 1/5 (4 cases) showed no improvement after treatment for 1 month and were switched to a glucocorticoid or combination treatment with a glucocorticoid, after which the disease was finally well-controlled. Side-effects of macrolide were rare. Based on this analysis, it is recommended that macrolides can be used as a first-line therapy in patients with mild COP. For patients with recurrent COP, it is suggested that macrolides should be used as an adjunctive therapy with other treatments, such as a glucocorticoid.

## Introduction

Organizing pneumonia (OP) is a pulmonary disorder that is associated with nonspecific clinical presentations, chest radiographic findings and pulmonary function results (1). OP is divided into primary and secondary OP based on etiology. Primary OP is referred to as cryptogenic organizing pneumonia (COP) and classified as an idiopathic interstitial pneumonia (1,2). Secondary OP is associated with a number of entities, including drugs, infections, malignancies, connective tissue diseases, organ transplantation, radiotherapy and the inhalation of harmful gases. COP mainly involves the alveoli, alveolar ducts and small airways; however, the lung interstitium may also be involved. It is considered as an inflammatory disease and diagnosed based on the clinical, radiographic and pathological findings following the exclusion of diseases associated with secondary OP (3). Glucocorticoids are effective in the treatment of COP. However, glucocorticoids usually take a longer time to take effect, and this results in severe side-effects.

Macrolides, a class of antimicrobial agents first isolated from *Streptomyces erythreus* more than five decades ago, are widely used to treat infectious respiratory diseases in humans (4). In addition to the anti-infectious effect, macrolides have been reported to have anti-inflammatory effects on certain inflammatory respiratory diseases, including asthma, bronchiectasis and cystic fibrosis (CF) (4). Studies have shown low-dose macrolides to be efficient in the treatment of COP (5–7). However, those studies are limited to case-based reports. There is a lack of controlled comprehensive clinical trials and definitive recommendations. The present study reports a case of COP that was effectively treated with azithromycin in combination with a glucocorticoid. A search of the literature was carried out in Medline, using the keywords ‘macrolide OR azithromycin OR erythromycin OR roxithromycin OR clarithromycin’ AND ‘cryptogenic organizing pneumonitis OR bronchiolitis obliterating organizing pneumonia’. A total of 35 articles were retrieved by December 2012. After excluding unrelated and repetitive articles, eight papers were selected for analysis. A review of 35 cases of OP/COP/bronchiolitis obliterans organizing pneumonia (BOOP) treated with macrolide antibiotics, from these papers and including the current case, was conducted.

## Case report

**Figure 1:**
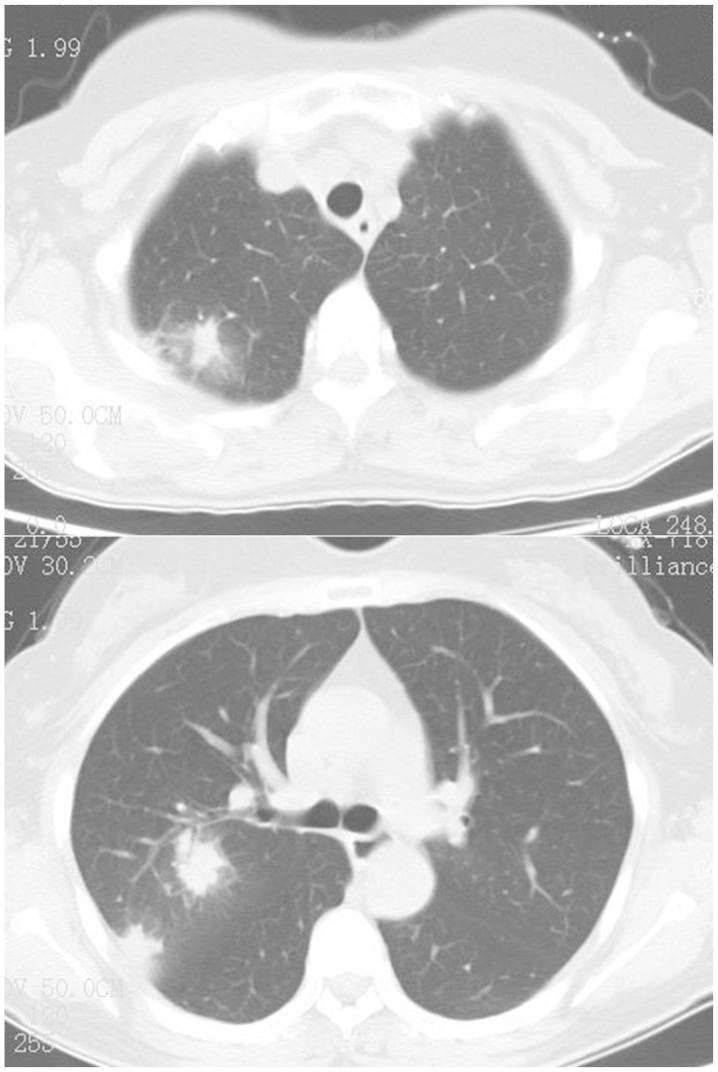
Initial chest radiography showing patchy, nodular consolidation in the right lung, and some localization in the subpleural area.

**Figure 2:**
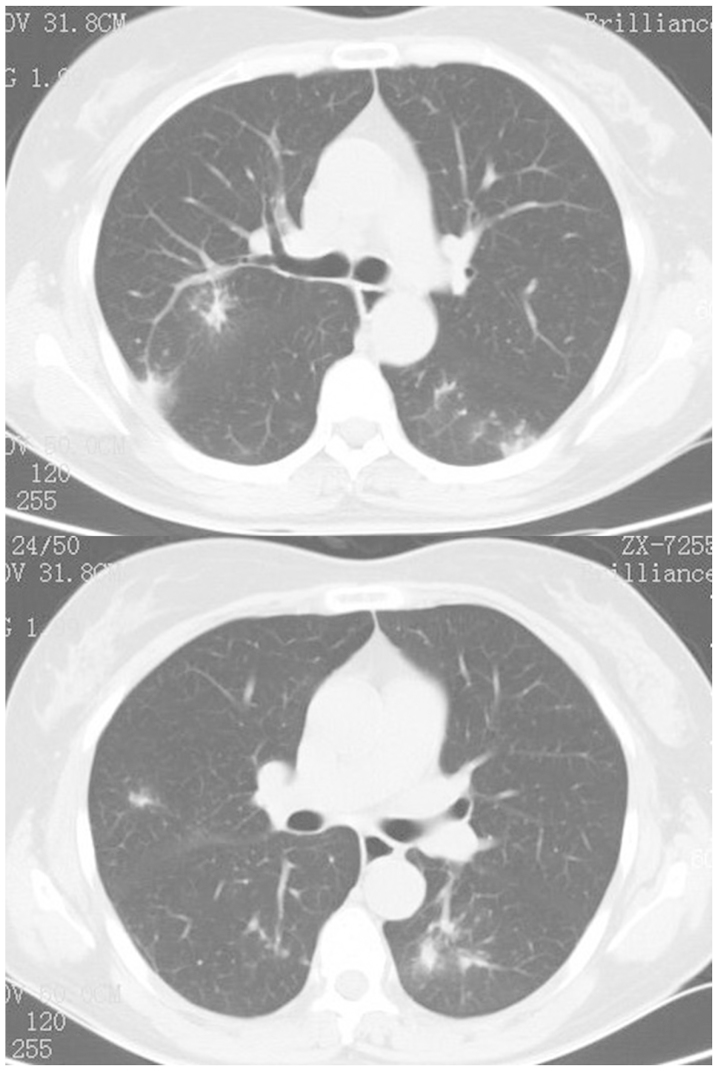
Chest radiography ten days after treatment showing patchy, nodular consolidation in the right lung and the lower lobe of the left lung.

**Figure 3:**
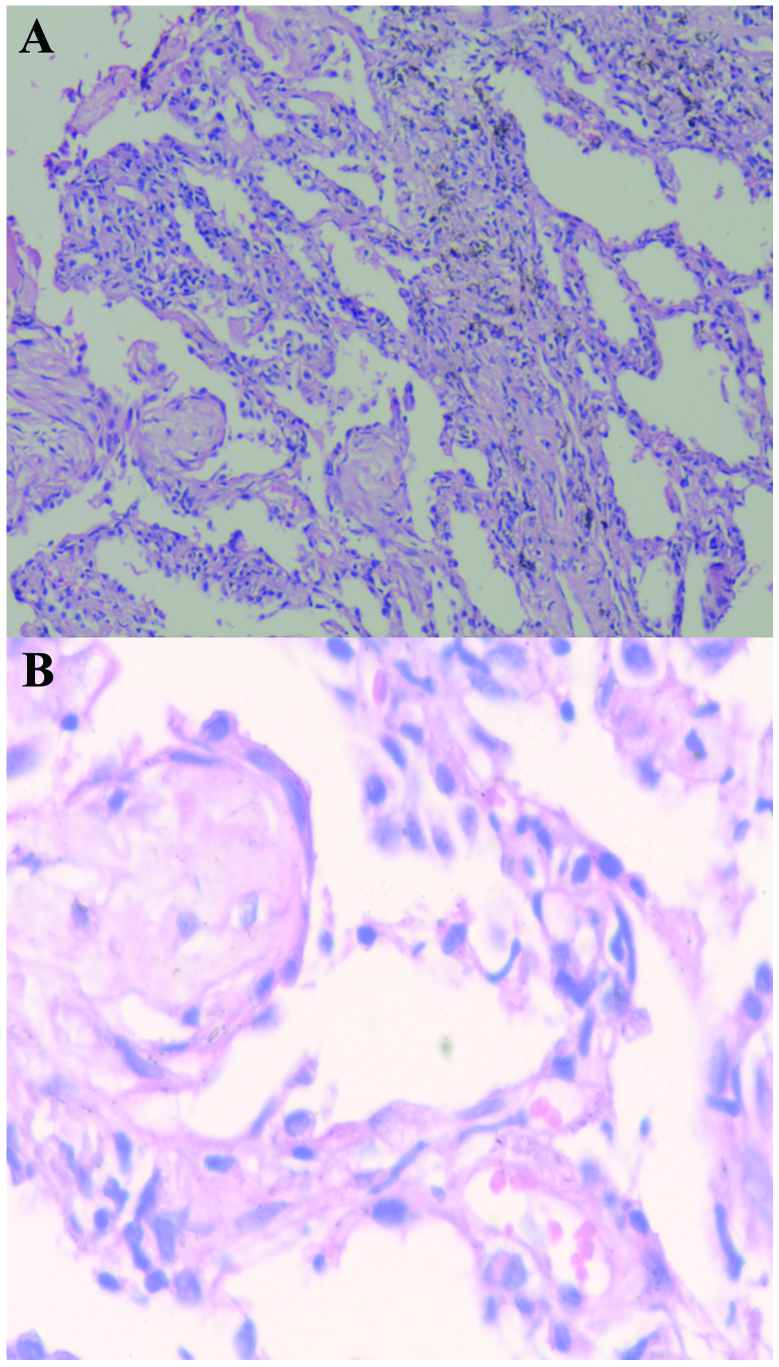
Histological changes in lung sections following lung biopsy. (A) Lower-power image showing patchy filling of the lung alveoli and respiratory bronchioles by loose plugs of granulation tissue (hematoxylin and eosin staining; magnification, ×10). (B) Higher-power image showing the swirled intra-airway plugs of fibroblasts and inflammatory cells compatible with organizing pneumonia (hematoxylin and eosin staining; magnification, ×40).

**Figure 4:**
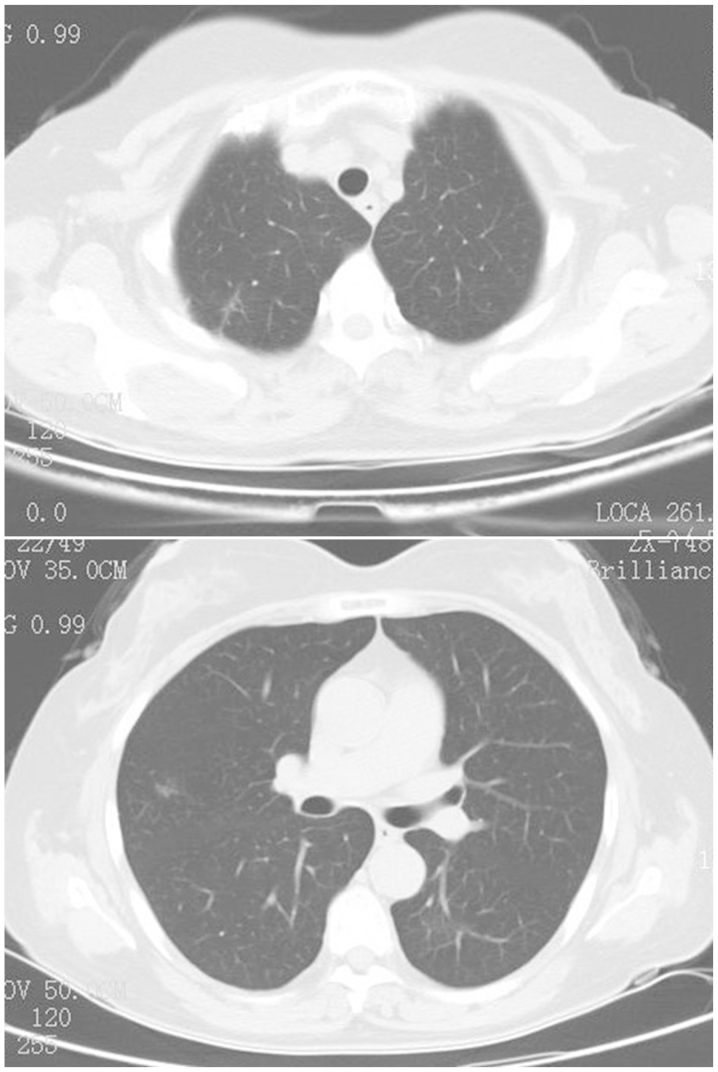
Chest radiography six months post-treatment showing a complete resolution of the previously observed bilateral patchy, nodular consolidations in the two lungs.

A 58 year-old female patient was admitted to the Affiliated Hospital of Ningbo University School of Medicine (Ningbo, China) with a nine-month history of concurrent cough, intermittent mild fever and fatigue. Prior to coming to the hospital, the patient had been treated with cephalosporins, quinolones and macrolides in other hospitals and the symptoms had not improved. The patient had been hypertensive for more than 10 years and was taking nifedipine orally to maintain a normal blood pressure. Physical examination revealed crackles in both lungs. The examination was otherwise normal, with no signs of connective tissue disorders. Chest computed tomography (CT) scanning showed bilateral nodular, patchy alveolar opacities, prominently in the right lung, and thickened pleura (Fig. 1). Laboratory tests showed a white blood cell (WBC) count of 8.3×10^9^/l, a neutrophil percentage of 63%, eosinophil percentage of 0.7%, red blood cell count of 4.46×10^12^/l, hemoglobin concentration of 116 g/l, platelet count of 369×10^9^/l, erythrocyte sedimentation rate of 112 mm/h and C-reactive protein level of 9.1 mg/l. Hepatic and renal function were normal. The serum anti-mycoplasma antibody titer was 1:80. The anti-double stranded DNA titer was 136 μg/l, the immunoglobulin E level was 41.58 kIU/l and the anti-paragonimiasis antibody test was negative. Immunological examinations were all normal. Perinuclear anti-neutrophil cytoplasmic antibody (P-ANCA) and cytoplasmic-ANCA tests were negative, and the tumor markers CA19-9, CA-125, carcinoembryonic antigen, neuron-specific enolase and CYFRA21-1 were normal. The blood gas pH was 7.41, the partial pressure of CO_2_ in arterial blood (PaCO_2_) was 39 mmHg, the PaO_2_ was 96 mmHg and the arterial oxygen saturation (SaO_2_) was 98%. Sputum culture was negative. Pulmonary function tests indicated small airway dysfunction. Bronchoscopy revealed a normal tracheal mucosa and lumen. A test of bronchoalveolar lavage fluid (BALF) showed no exfoliated cells. The cell types were 2.4% neutrophils, 0% eosinophilic granulocytes, 82.3% macrophages and 15.3% lymphocytes. Transbronchial needle aspiration did not detect cancer cells. The patient was initially considered to have community acquired pneumonia and was treated with moxifloxacin (0.4 g, intravenous drip, daily). Ten days later, a chest CT scan demonstrated that the lesions on the right upper lung were slightly absorbed; however, there were new nodular and patchy ground glass opacities in the left lung (Fig. 2). A percutaneous lung biopsy in the lower left lung was performed. The pathologic examination identified typical characteristics of OP. The alveoli and alveolar ducts were filled with plugs of granulation tissue composed of fibroblasts. Chronic inflammatory cell infiltration and a few scattered giant cells were found in these tissue sections (Fig. 3). The patient was treated with 0.75 mg/kg/day of prednisone orally. Two weeks later, respiratory symptoms disappeared and chest radiographic abnormalities were improved. After another 2 weeks of prednisone treatment, the patient complained of gastrointestinal discomfort. Thus, 500 mg/day of azithromycin was added to the treatment, and the dose of prednisone was gradually reduced. Three months later, prednisone was completely withdrawn. The patient was continually given azithromycin at 500 mg/day for another three months. A chest CT scan showed a complete resolution of the previous bilateral patchy, nodular consolidations in both lungs (Fig. 4). The patient was followed-up for one year, and the disease did not relapse. Informed consent was obtained from the patient.

## Discussion

COP is a type of organizing pneumonia without the presence of evident pathogens, such as infection, or other associated diseases, such as connective tissue disease. COP is commonly observed in the non-smoking population and predominantly in female patients. In the present review of 35 cases of OP/COP/BOOP, there were 12 male and 23 female patients. Their ages were between 13 and 83 years. Radzikowska *et al* (6) reported that 10/12 patients were non-smokers, and 9/12 were female patients. In the cases covered in the current review, there were two smokers, four ex-smokers and 14 non-smokers; the smoking history of the remaining nine patients was not mentioned in the papers. There were 23 cases of primary COP and six cases of secondary COP. Three cases were associated with radiotherapy, and the other three cases were associated with amiodarone, chemotherapy and bone marrow transplantation, respectively. None of them had family history of COP.

COP patients usually do not show unique clinical presentations. Among the 35 reviewed cases, 29 patients had certain types of symptoms; the symptoms of the other six patients were not specified. The most common symptoms were cough (24/29), shortness of breath (18/29), light fever (16/29), fatigue (15/29), weight loss (11/29) and night sweats (6/29; Table I). The complications included pneumothorax in one case (5), and mediastinal emphysema and pneumothorax in one case (4). The interval time from onset of symptoms to diagnosis was variable, with the longest being a 5-year medical history for a 60-year-old female patient. During these 5 years, the patient repeatedly presented with fever, cough and shortness of breath. The patient was initially diagnosed with asthma, and was pathologically diagnosed with COP through percutaneous lung biopsy (7). Physical examinations were usually normal in these patients. Nine out of 29 patients had lung wheezing, and two had lung crackles. The majority of the patients had mild illnesses; only one patient had acute onset of illness, and the illness rapidly progressed following prednisone treatment, and had complications of pneumomediastinum and pneumothorax. The disease was improved after the patient was given macrolide in combination with methylprednisolone and cyclosporine (8).

The majority of the patients had abnormal chest radiographic findings. The most common manifestations were multiple patchy opacities (21/29), air bronchogram (16/29), multiple lung nodules (5/29), ground glass opacity (5/29), multiple consolidation (2/29) and single consolidation (3/29; Table I). The most common feature in the chest radiography of patients with COP was a migratory pattern. It existed in 9/12 patients in a single study (75%) (6). In the present review, it was observed in 37.9% of patients (11/29). The majority of the lesions involved bilateral lungs; both right and left lungs were involved in 28 cases. Pleural effusion and lymphadenopathy were rare. The radiographic findings were variable in OP, and the differentiation of OP from other pulmonary diseases is challenging. Lung biopsy is extremely important to the diagnosis of OP. All 35 patients were diagnosed by either cytology or pathology. Transbronchial lung biopsy (TBLB) was the most frequently used technique, and it had the highest diagnostic value among all methods. Thirteen out of 15 patients were diagnosed through TBLB. TBLB failed to diagnose the remaining two cases, which were diagnosed through thoracoscopy. Four cases were diagnosed through percutaneous lung biopsy, one of them was confirmed at the second examination. Seven patients were confirmed by surgery and three were confirmed by thoracoscopy. Surgical intervention is not recommended as the first choice due to its invasiveness. TBLB is strongly recommended as a routine diagnostic technique for COP patients.

COP is considered to be an inflammatory disease. BALF cytological examination demonstrated increased lymphocyte counts, decreased CD4/CD8 ratio and increased CD8^+^CD11b^−^ cell levels (9). In this review, BALF cytological classification was dominated by lymphocytes (8/16; 50%). Cai *et al* (10) observed that the cytokines interleukin (IL)-6, -8, and -10, interferon-γ-inducible protein 10, tumor necrosis factor (TNF)-α, soluble TNF receptor 1 (STNFR1) and STNFR2 in BALF were significantly increased in COP patients, and macrolides significantly decreased their levels. The proportions of lymphocytes, neutrophils and CD8^+^CD11b^−^ cells were significantly decreased following macrolide treatment (10). This suggests that increased lymphocyte counts and numbers of CD8^+^CD11b^−^ cells in BALF have diagnostic implications for COP.

Glucocorticoids are traditionally a first-line agent for treating COP. The majority of patients respond well to treatment (3). However, one study reported that 10–15% of patients were resistant to treatment and the disease progressed rapidly (11). A total of 13–58% of patients had recurrent disease during the reduction of glucocorticoids or following drug discontinuation (11). Macrolides, as regulators of immune responses, have been widely used in bronchial asthma, bronchiectasis, diffuse panbronchiolitis and other diseases. They have also been reported to effectively treat COP (6–8,10,12–14). In the current review, all 35 patients received macrolide treatment, including six cases with unknown clinical efficacy. The efficacy was assessed in the remaining 29 patients. Twenty patients initially received macrolide as a single agent. Four-fifths of the patients (16 cases) were cured with the medication after 3–14 months; however, the improvement took a longer time than that of glucocorticoids. It usually took 2–3 weeks for symptom improvement and 1 month for radiological improvement on chest images. One-fifth of the patients (four cases) had no improvement following macrolide treatment for 1 month and had to switch to glucocorticoids or a combination treatment with glucocorticoids. After that, the disease was well-controlled. The most commonly used macrolide was clarithromycin (19 cases), the second was erythromycin (seven cases), and the next was azithromycin (two cases). The type of macrolide used was undocumented in one case. Nine patients were later treated with macrolide due to poor efficacy or side-effects from glucocorticoids. Following the addition of macrolide, the symptoms in these patients were significantly improved (Table II). There was one patient with severe COP in whom the disease continued to deteriorate during glucocorticoid treatment. Following treatment with a combination of cytotoxic drugs and macrolide, the disease attenuated rapidly (8). Side-effects of macrolide treatment were rare. There was only one patient who had a skin rash resulting from clarithromycin, and later this drug was stopped (14).

The molecular mechanism of macrolides in the treatment of COP is not fully understood. Pathologically, COP is mainly manifested as a polypoid granulation hyperplasia in the small airway and alveolar respiratory bronchioles. The granulation tissue is composed of fibroblasts/myofibroblasts, infiltrated with inflammatory cells, including monocytes, macrophages, mast cells, a few eosinophils and neutrophils, particularly in the early stage of the disease. Studies have shown that macrolides exhibit anti-inflammatory effects (15–20). They have been widely used in a variety of acute and chronic respiratory diseases and are effective in treating these diseases. Kudoh (21) suggested that erythromycin significantly improved the survival rate of patients with diffuse panbronchiolitis. Itkin and Menzel (22) demonstrated that macrolides could lower airway hyper-responsiveness and reduce the amount of corticosteroid used in patients with asthma. These lines of evidence support the anti-inflammatory effect of macrolides in the treatment of COP. The efficacy of a macrolide not only relies on its treatment time but also its molecular structure. The 14- and 15-membered ring antibiotics have been reported to have anti-inflammatory effects, and these antibiotics include macrolides such as erythromycin, clarithromycin and azithromycin (23).

Based on the present analysis, it is recommended that macrolides may be used as first-line therapeutic agents in patients with mild COP. For patients with recurrent COP or during the reduction of glucocorticoid dosing, it is suggested that macrolides may be combined with steroids as an adjuvant therapy. Prior to any definitive recommendations being made, large-scale randomized controlled clinical trials are required.

## Figures and Tables

**Table I:** Clinical characteristics of patients with COP.

Characteristics	No. of cases	Percentage of total^a^
Gender		
Male	12	
Female	23	
Age		
13–83 years	35	
Smoking		
Never	14/20	70
Ex-smoker	4/20	20
Smoker	2/20	10
COP		
Primary	24	82.8
Secondary	5	17.2
Symptoms		
Cough	24	82.8
Dyspnea	18	62.1
Low-grade fever	16	55.2
Weight loss	11	37.9
Fatigue	15	51.7
Night sweats	6	20.7
Lung signs		
Crackles	2	6.9
Wheezing	9	31
Chest radiograph		
Normal		6
Abnormal		29
Patchy consolidation	21	72.4
Bilateral	28	96.6
Multiple nodules	5	17.2
Pleural effusion	2	6.9
Migratory lesions	11	37.9
Ground glass opacities	5	17.2
Air bronchogram	16	55.2
Lung biopsy		
TBLB	13	44.8
Thoracoscopy	5	17.3
Percutaneous lung biopsy	4	13.8
Surgery	7	24.1
Macrolide		
Alone	5	82.8
Adjunctive	24	17.2
Efficacy		
Effective	24	82.8
Invalid	5	17.2

COP, cryptogenic organizing pneumonia; TBLB, transbronchial lung biopsy.

a n=29 with known outcome; n=20 with known smoking status.

**Table II:** Cases of OP/COP/BOOP treated with macrolide.

Reference	Year	No. of patients	Gender	Age (years)	Diagnosis	Macrolide	Regimen	Efficacy
Cai *et al* (10)	2013	6	1F/5M	64±2	BOOP	CAM or AZM	Unknown	Unknown
Chang *et al* (5)	2012	1	F	37	COP	CAM	Adjunctive	Effective
Vaz *et al* (7)	2011	1	F	60	COP	AZM	Adjunctive	Effective
Lee et al (8)	2011	1	F	38	COP	Macrolide	Adjunctive	Effective
Radzikowska *et al* (6)	2008	12	9F/3M	44–71	OP	CAM	Single	Effective 9; invalid 3
Stover *et al* (14)	2005	6	M	72	OP	CAM	Single	Effective
			M	76	OP	CAM	Single	Effective
			F	65	OP	CAM	Single	Invalid
			F	56	BOOP	CAM	Single	Effective
			F	62	BOOP	CAM	Single	Effective
			M	67	BOOP	CAM	Single	Invalid
Ishii *et al* (13)	2000	1	M	13	BOOP	EM	Adjunctive	Effective
Ichikawa *et al* (12)	1993	6		52±21.8				
			F	47	BOOP	EM	Single	Effective
			F	57	BOOP	EM	Single	Effective
			F	64	BOOP	EM	Single	Effective
			F	83	BOOP	EM	Single	Effective
			F	43	BOOP	EM	Single	Effective
			F	18	BOOP	EM	Single	Effective
Present case	2013	1	F	58	COP	AZM	Adjunctive	Effective

F, female; M, male; OP, organizing pneumonia; COP, cryptogenic organizing pneumonia; BOOP, bronchiolitis obliterans with organizing pneumonia. CAM, clarithromycin; AZM, azithromycin; EM, erythromycin.
